# Prioritization of Resilience Initiatives for Climate‐Related Disasters in the Metropolitan City of Venice

**DOI:** 10.1111/risa.13823

**Published:** 2021-09-17

**Authors:** Marta Bonato, Beatrice Sambo, Anna Sperotto, James H. Lambert, Igor Linkov, Andrea Critto, Silvia Torresan, Antonio Marcomini

**Affiliations:** ^1^ Fondazione Centro Euro‐Mediterraneo sui Cambiamenti Climatici (Fondazione CMCC) c/via Augusto Imperatore 16 Lecce 73100 Italy; ^2^ University of Ca’ Foscari Via Torino 155 Venezia Mestre 30170 Italy; ^3^ Helmholtz‐Centre for Environmental Research ‐ UFZ 15 Permoserstraße Leipzig 04318 Germany; ^4^ Basque Centre for Climate Change (BC3) Scientific Campus of the University of the Basque Country Building 1, Barrio Sarriena 48940, Leioa Bizkaia Spain; ^5^ University of Virginia Charlottesville VA USA; ^6^ Engineer Research and Development Center, U.S. Army Corps of Engineers Concord MA USA; ^7^ Carnegie Mellon University Pittsburgh PA USA

**Keywords:** Climate change, critical functions, risk management, scenario‐based preferences, systems engineering, uncertainty analysis, Venice

## Abstract

Increases in the magnitude and frequency of climate and other disruptive factors are placing environmental, economic, and social stresses on coastal systems. This is further exacerbated by land use transformations, urbanization, over‐tourism, sociopolitical tensions, technological innovations, among others. A scenario‐informed multicriteria decision analysis (MCDA) was applied in the Metropolitan City of Venice integrating qualitative (i.e., local stakeholder preferences) and quantitative information (i.e., climate‐change projections) with the aim of enhancing system resilience to multiple climate‐related threats. As part of this analysis, different groups of local stakeholders (e.g., local authorities, civil protection agencies, SMEs, NGOs) were asked to identify critical functions that needs to be sustained. Various policy initiatives were considered to support these critical functions. The MCDA was used to rank the initiatives across several scenarios describing main climate threats (e.g., storm surges, floods, heatwaves, drought). We found that many climate change scenarios were considered to be disruptive to stakeholders and influence alternative ranking. The management alternatives acting on physical domain generally enhance resilience across just a few scenarios while cognitive and informative initiatives provided resilience enhancement across most scenarios considered. With uncertainty of multiple stressors along with projected climate variability, a portfolio of cognitive and physical initiatives is recommended to enhance resilience.

## 1 INTRODUCTION

Climate change is compounding with various threats both to natural and human coastal systems (Intergovernmental Panel on Climate Change [IPCC], 2021), by increasing the frequency, duration, and intensity of many types of climate‐related extreme events (European Environment Agency [EEA], 2017; IPCC, 2021, 2014). Extreme events can act as triggering factors for disasters (IPCC, 2021). According to UNDRR (United Nations Office for Disaster Risk Reduction), over the last 20 years 90% of major disasters have been caused mainly by floods, storms, heatwaves, droughts, and other climate‐related extreme events (Wallemacq & Below, 2015). Given the importance of understanding the relationship between climate change and other stressors, fostering a coherent integration of Climate Change Adaptation (CCA) and Disaster Risk Reduction (DRR) concepts is becoming a global and European priority. The current European Union (EU) development policy context (i.e., the adoption of the 2030 Agenda for Sustainable Development (United Nations [UN], 2015), the Paris Agreement on Climate Change (United Nations Framework Convention on Climate Change [UNFCCC], 2015), the Sendai Framework (UNDRR, 2015)) recognizes how a stronger integration of CCA and DRR could help in addressing important EU Societal Challenges (e.g., climate action), supporting the achievement of multiple Sustainable Development Goals (SDGs) (UN, 2015) (e.g., SDG6, SDG7, SDG9, SDG13, SDG14, and SDG15) and disaster risk reduction targets (UNDRR, 2015).

Through the concept of resilience, we are able to enhance the integration of CCA and DDR (Howes, 2015), by incorporating traditional risk assessment into a wider framework that embraces strategies of adaptation to improve disaster risk management (see, e.g. Bostick, Connelly, Lambert, & Linkov, 2018; Bostick, Holzer, & Sarkani, 2017; Connelly, Lambert, & Thekdi, 2016; Donnan et al., 2020; Hamilton, Lambert, Keisler, Holcomb, & Linkov, 2013; Hamilton, Lambert, & Valverde, 2015; Karvetski & Lambert, 2012; Karvetski, Lambert, & Linkov, 2009; Karvetski, Lambert, Keisler, & Linkov, 2011; Karvetski, Lambert, Keisler, Sexauer, & Linkov, 2011; Lambert et al., 2012; Lambert, Wu, You, Clarens, & Smith, 2013; Parlak, Lambert, Guterbock, & Clements, 2012; Quenum, Thorisson, Wu, & Lambert, 2019; Schroeder & Lambert, 2011; Thorisson, Lambert, Cardenas, & Linkov, 2017; You, Connelly, Lambert, & Clarens, 2014; You, Lambert, Clarens, & McFarlane, 2014). Various definitions of resilience (Fox‐Lent, Bates, & Linkov, 2015) have been explored in several and distinct contexts, from ecology (Gunderson, Allen, & Holling, 2012; Holling, 1973; Walker, Holling, Carpenter, & Kinzig, 2004), to engineering (Ganin et al., 2016; Holling, 1996), to disaster risk reduction (Rose, 2004). In the disaster risk reduction context, resilience is defined as “*the ability of a system, community or society exposed to hazards to resist, absorb, accommodate, adapt to, transform and recover from the effects of a hazard in a timely and efficient manner, including through the preservation and restoration of its essential basic structures and functions through risk management”* (UNDRR, 2009). Several efforts (Bostick et al., 2017; Hosseini, Barker, & Ramirez‐Marquez, 2016; Linkov et al., 2018; Linkov et al., 2014) identify resilience as “emergent system property” arising from the complex interactions among components of the system under analysis including the physical components and the social, institutional, and informational services that enable their effective use.

Especially in the context of climate related‐disasters, characterized by low probability, high consequence risks, and uncertainty, this concept can be particularly useful to bound the system under study, identifying critical system functionalities relevant for stakeholders and add the consideration of longer‐term horizons (Taarup‐Esbensen, 2020) in the risk reduction and adaptation processes.

In other words, resilience offers the opportunity of bringing a different perspective that otherwise may be missed by traditional risk analysis approaches: the ability to understand the capacity of system to recover from a massive external shock (Linkov, Trump, & Fox‐Lent, 2016). While it is inherently impossible to foresee a highly uncertain and infinitely diverse future, resilience can improve system capacity to quickly cope and adapt to multiple climate change related stressors (Terzi et al., 2019).

On the other hand, a robust resilience analysis cannot be performed aside from the consideration of the type of events which may potentially occur. In this sense risk assessment can supplement resilience analysis with new insights about unknown and potentially surprising types of events as well as “cause–effect” relationships between stressors in a way that targeted and more efficient measures can be proposed (Aven, 2017).

This sort of complementary relationship between resilience and risk has been recognized and discussed by several authors from both fields (Linkov et al., 2016; Park, Seager, Rao, Convertino, & Linkov, 2013; Trump, Florin, & Linkov, 2018). While these studies explored the topic at a conceptual level, this article finally shows how such integration of risk assessment into resilience analysis can be operationalized in practice, specifically merging together an expert‐based assessment of climate change risks and local stakeholders’ preferences and choices regarding the system under analysis.

For this purpose, the scenario‐based multicriteria decision analysis (MCDA) methodology initially developed by Linkov and Lambert (Bostick et al., 2017; Fox‐Lent, & Linkov, 2018; Linkov et al., 2018) was further adapted to permit the integration of bottom‐up qualitative information (i.e., goals, alternatives and constraints of local actors), typically employed in resilience analysis, with quantitative metrics derived by top‐down climate change risk assessment into a unique assessment framework. Given its adaptive and iterative nature, we employed the proposed approach to pursue the following research objectives: (i) explore local actors’ priorities in terms of critical components of the system that should be protected and the typology of risk management measures to be implemented; (ii) assess how such priorities are likely to be disrupted/impacted by foreseen climate change scenarios; (iii) reprioritize proposed initiatives accordingly in order to identify the best set of measures to enhance the overall resilience of the system toward multiple climate‐related disasters. The methodology was implemented and tested with the local actors of the Metropolitan City of Venice in the frame of the BRIDGE project, a bilateral cooperation between Italy and the United States funded by the Italian Ministry of Foreign Affairs and International Cooperation. The area is extremely vulnerable to different type of climate related extreme events (Barbi, Formentini, Monai, Rech, & Zardini, 2007; Biolchi et al., 2019; Ferrarin, Chiggiato, Schroeder, & Zaggia, 2019; Lionello et al., 2020), due to its geographical, geomorphological, and climatic characteristics (Međugorac, Pasarić, & Güttler, 2021). Such kind of events in the last decade have increased in frequency and intensity (Caporalini, Deuss, & Godlewski, 2020; Lionello et al., 2020; Međugorac et al., 2021; Morucci, Coraci, Crosato, & Ferla, 2020), calling urgently for the implementation of risk management strategies and the adoption of a tailored climate change adaptation plan compatible with the economic development of the area and shared by the numerous actors and visions involved.

## 2 METHODOLOGY

The scenario‐informed multicriteria methodology proposed aims at the integration of different data (i.e., priorities on critical functions and risk management initiatives and climate‐related scenarios) to analyze the interconnectivity of different domains and stages of coastal resilience and support disaster risk management. Given the specificities of the analyzed case study and the multiplicity of involved interests (see Section 2.1), the methodology was codeveloped step by step with local actors involved at different stages of the disaster risk management cycle (see Section 2.2). A structured participative process was implemented starting with stakeholder's analysis, a first round of individual semistructured interviews, and stakeholders’ selection. Based on this, a small group of stakeholders was invited to take part in a workshop which in addition to providing them with the most up‐to date climate information and scenarios expected in the case study allowed to elicit their perspectives and preferences on different input data (i.e. the critical functions, risk management initiatives), as well as allowed to collect the relevance values necessary for the different steps of the methodology (see Section 2.3).

### 2.1 Resilience Issues and Challenges in Venice

**Fig 1:**
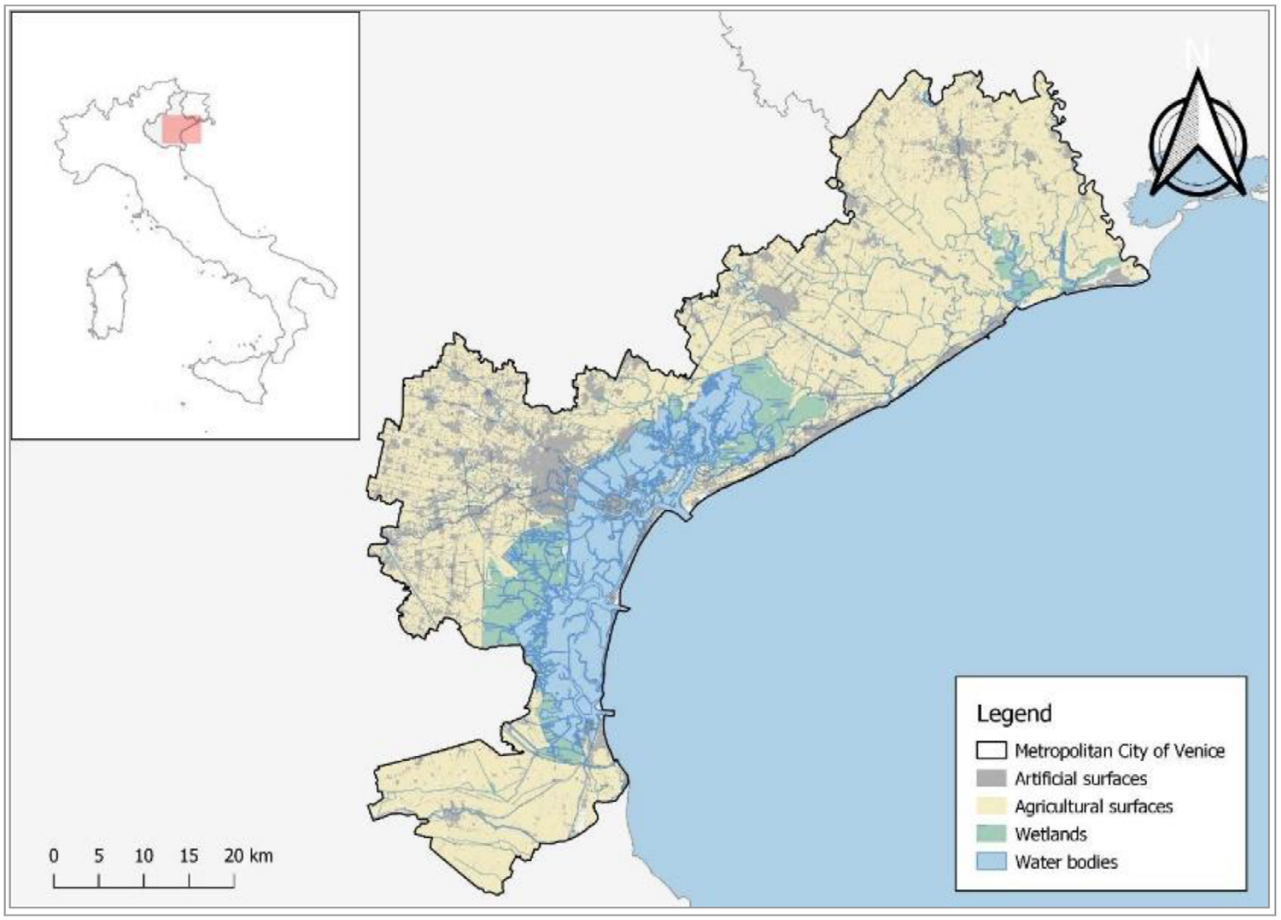
Case study area.

The case study is represented by the Metropolitan City of Venice and its lagoon located in the North‐East of Italy, along the Adriatic coast (Fig. 1).

It represents a coastal‐urban system that is facing multiple challenges related both to global change phenomena and socioeconomic dynamics. The Metropolitan City of Venice is a densely urbanized and populated area with 842 942 inhabitants in 2020 (Italian National Institute of Statistics [ISTAT], 2021), of which 255 609 reside in the municipality of Venice. A variety of economic activities are conducted in this area, such as fishery, aquaculture, agriculture, and maritime shipping. Moreover, a fundamental role is played by tourism: Venice itself is one of the most visited destinations in Italy and Europe with 12 million touristic presence in 2019 (Regione Veneto, 2021). Due to its natural characteristics, there is also a need to ensure environmental protection in this area; the Venice lagoon is itself a UNESCO site since 1987 where several areas are safeguarded at regional or national level or under Natura 2000 protection (Regione Veneto, 2021). The climatic and geographic conditions contribute to make the Metropolitan City of Venice naturally vulnerable to climate related extreme events. In recent years, frequent high tides, pluvial floods, windstorms, drought, and heat waves have become more frequent and intense (Caporalini et al., 2020; Gallina et al., 2020; Lionello et al., 2020) leading to both significant environmental and economic losses. In 2007 a severe pluvial flood event hit the municipality of Venice with more than 260.4 mm of precipitation in 24 hours (Barbi et al., 2007), causing widespread flooding in urban and agricultural areas close to the Venice Lagoon; the main damages were recorded in Mestre and in the nearby agricultural areas located among the coast (Rossa et al., 2010). In 2015, a tornado struck the area of Riviera del Brenta causing damage to about 320 residential buildings (Zanini, Hofer, Faleschini, & Pellegrino, 2017). In October 2018, the Vaia storm hit the Veneto region with heavy precipitation and strong winds, causing damage to forests and to the boundary areas. Finally, the city of Venice is periodically subjected to sudden and frequent high tides. In 2019, due to a simultaneous occurrence of extreme high tide, strong Scirocco winds, and heavy rainfalls, the water level reached the height of 187 cm, becoming the second highest recorded high water and causing the flooding of 88% of the city (Ferrarin et al., 2019). The extreme high tide caused significant damage to residential structures, economic activities, cultural heritages, transport facilities and, unfortunately, also caused the loss of one human life. Climate change, which affects the frequency and intensity of extreme events, tends to increase the occurrence of weather‐related disasters (World Economic Forum [WEF], 2021). Accordingly, there is the need to plan for adaptation by implementing a set of risk management initiatives to increase the overall resilience of the Metropolitan City of Venice toward disasters that involve climate and the variety of other stressors.

### 2.2 Stakeholders

The scenario‐based methodology presented in this article took advantage of a strong engagement of local stakeholders of the Metropolitan City of Venice. Stakeholders play an important role by providing their perspectives and identifying needs, which is useful to improve the decision‐making process. According to Bostick et al. (2017), the participative process is essential for the implementation of the methodology for building resilience in coastal areas and for the codevelopment of information, which can be practically used for climate change adaptation and disaster risk management planning. The engagement process should be as inclusive as possible, involving actors covering different sectors and different stages and domain of disaster risks and resilience management. This ensures that personal perspectives and bias are not going to outweigh the final results, that needs and perspectives of minority groups are taken into account (Luyet, Schlaepfer, Parlange, & Buttler, 2012), and that different systems connection and interdependencies are fully understood (Walker, Anderies, Kinzig, & Ryan, 2006).

Stakeholders to be involved were selected among an initial list of thirty authorities and local actors (e.g. national, regional and local authorities, research institutions, regional meteorological offices, environmental protection agencies, nongovernmental organizations [NGOs] and small and medium‐sized enterprises [SMEs], sector representatives) which actively work at different stages of disaster risk management and represent different fields of expertise (e.g. civil protection, environmental protection, cultural heritage management, primary, secondary, and tertiary sectors). A first contact with local stakeholders was established by means of semistructured telephone interviews aimed at understanding what types of data they were using, and what type of works they were carrying out in the field of disaster risk management. After this first semistructured interview, a small group of stakeholders coming from different levels of institutions and organizations were selected and invited to take part in a local workshop called “Building the Resilience of the Metropolitan City of Venice and its Lagoon to Disasters,” held on October 30, 2019. Although attention was paid to invite a group of local actors as heterogenous as possible, in the end only 15 participated. As described on Table I, manufactured, social and natural sectors are well represented by participants, while cultural and economic sectors are slightly less represented. Through focus groups discussions and an individual questionnaire, we collected local actors’ preferences on input data (e.g., the critical functions, risk management initiatives and scenarios). Such results have been elaborated after the workshop and included in the analysis as described in the following Section 2.3.

**Table I:** List of Institutions and Organizations Participating in the 2019 Workshop with their Sector(s) of Expertise

Level	Institution	No.	Expertise Sector(s)
National authorities	Italian Ministry of Transports and Infrastructures	2	Manufactured, Economic
	Italian Institute for Environmental Protection and Research (ISPRA)	2	Natural, Social
Regional authorities	Regional Civil Protection Agency	1	Social, Manufactured
	Regional Agency for Environmental Prevention and Protection (ARPAV)‐Metereological Service	1	Natural
Local authorities	Venice Municipality‐Environmental Department	1	Natural
	Metropolitan City of Venice‐ Environmental, Civil Protection and Agriculture Department	1	Natural, Social, Manufactured, Economic
Independent authorities	Consortium for the protection of the Venetian lagoon (Consorzio Venezia Nuova)	1	Manufactured, Natural, Economic
	Veneto Orientale Reclamation Consortium	1	Manufactured, Natural, Economic
Research institutions	Consortium for Coordination of Research Activities concerning the Venice Lagoon system (CORILA)	1	Natural, Cultural
	Euro‐Mediterranean Centre on Climate Change (CMCC)	1	Natural, Social
Parks	Lega Italiana Protezione Uccelli Italian (LIPU)	1	Natural
NGOs	Venice Resilience Lab	1	Cultural, Social, Natural
	We are here Venice	1	Cultural, Social, Natural

### 2.3 The Methodological Framework for Resilience Assessment

**Fig 2:**
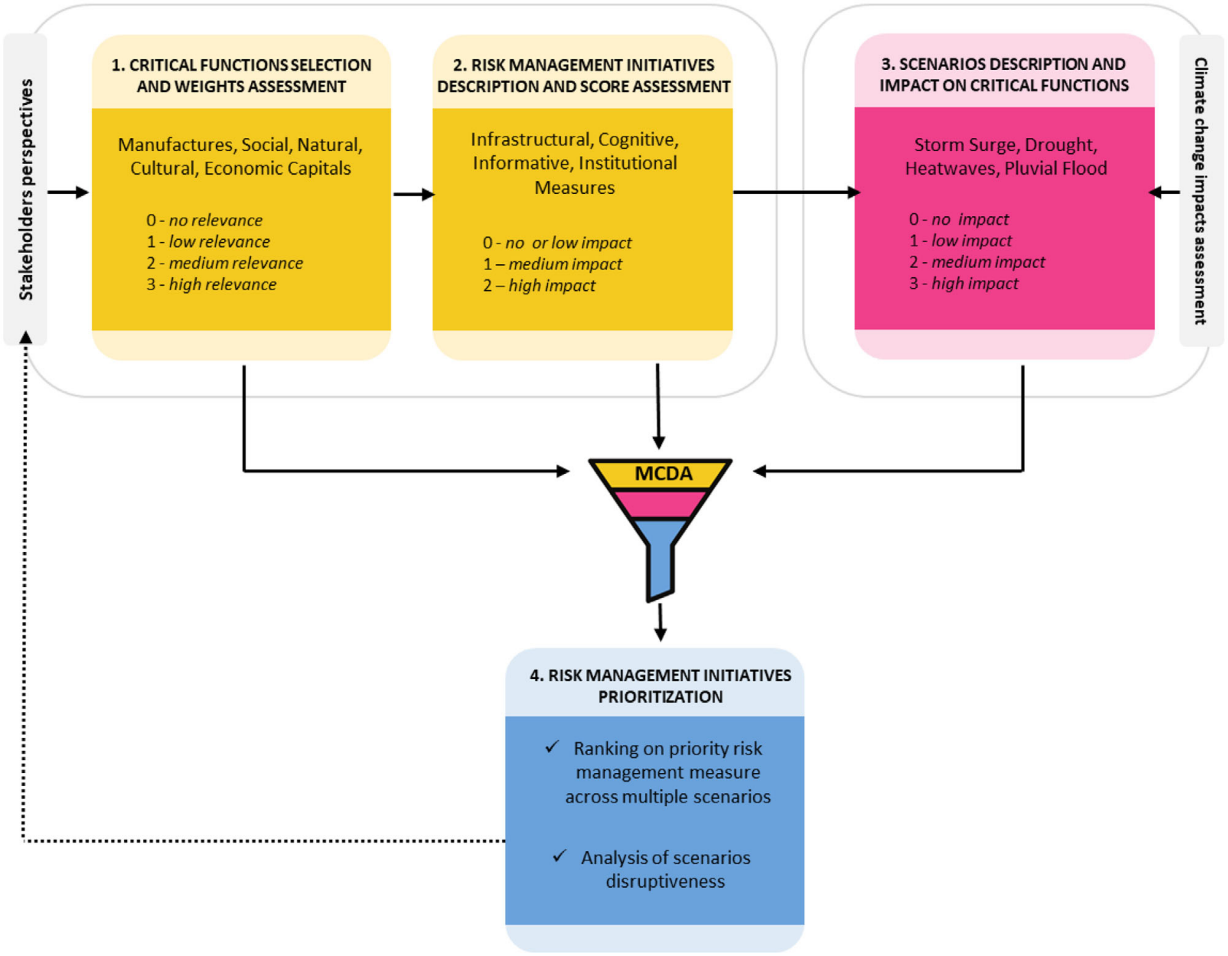
Methodological framework for resilience assessment in the Metropolitan City of Venice (Adapted from Bostick et al., 2017; Linkov et al., 2018).

Environmental decisions are complex and built upon multidisciplinary approaches and knowledge. Considering that various types of decisionmakers are relying on the integration of experimental tests, models, and tools, it is necessary to find a methodology flexible enough to incorporate the huge amount of data and heterogenous information available (Huang, Keisler, & Linkov, 2011). Accordingly, in this article a scenario‐informed multicriteria methodology is applied to support disaster risk management, exploiting available data to analyze the interconnectivity of different domains and stages of coastal resilience. MCDA and scenario planning have been jointly used to bring together different local stakeholders to discuss and plan adaptation to climate‐related extreme events. The incorporation of *scenario‐based preferences* to risk analysis is introduced by Schroeder and Lambert (2011), while subsequent efforts extended the approach to both risk and *resilience* (e.g., Thorisson et al., 2017). The approach of this article (Fig. 2) is based on the resilience assessment methodology originally developed by Fox‐Lent and Linkov (2018) and Linkov et al. (2018), which is here adapted to include top‐down quantitative information and assessments (e.g., climate change projections coming from regional climate models and impact analysis). These are necessary to characterize future climate change scenarios and related risks for the coastal area of interest, thus providing the basis for the prioritization of a set of risk management initiatives against a set of “plausible futures” based on assumptions about climatic and socioeconomic development.

The methodology applied relies on different iterative steps (Fig. 2). The first requires the identification of key critical functions (i.e., subsystems and processes that are affected by climate related‐extreme events) for the system under analysis, which will form the basis for risk management initiatives comparison. Later, the relative importance of each critical function within the system (e.g., *no*, *low*, *medium*, and *high* relevance) is assessed by the stakeholders and subsequently the qualitative relevance value of the critical functions is converted to a relevance value weight (Supporting Information, Appendix I).

**Fig 3:**
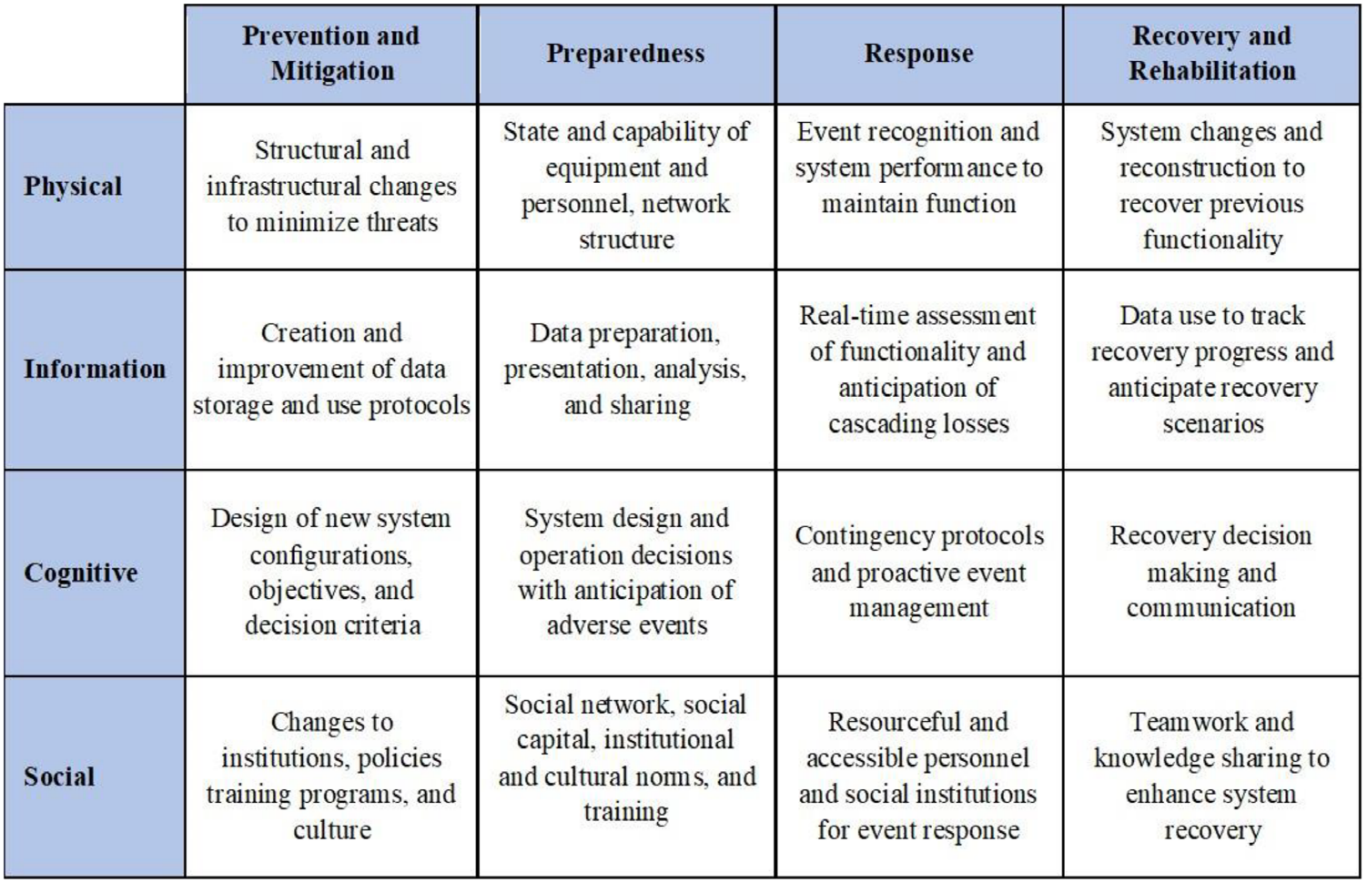
Resilience matrix for characterizing the dimensions of initiatives for coastal systems. Adapted from Fox‐Lent et al., 2015 and Bostick et al. (2017).

In the second step (Fig. 2) a set of risk management initiatives aimed at enhancing the overall resilience of the system to climate‐related extreme events are selected and proposed for prioritization. Risk management initiatives can involve the allocation of resources, policies, structural and nonstructural interventions encompassing different risk‐management stages and belong to several domains as described in Fig. 3.

Following Thorisson et al. (2017), for each risk management initiative identified, the impact on each of the critical functions previously identified is assessed by local stakeholders by assigning an impact value (e.g., *no*, *low*, *medium*, and *high* impact). The impact value score indicates how much the project initiative could impact the critical function: a low impact value score means that the project initiative has low or no impact on that particular critical function, while a higher value means that the initiative has a significant impact on the critical function. Also in this case, the qualitative impact value of the risk management initiatives on the critical functions is subsequently converted in a quantitative score (Supporting Information, Appendix I). A multicriteria value function (Supporting Information, Appendix I, Equation A1) is then used to incorporate the weights representing the relative importance of the critical functions and the scores representing the impact of the risk management initiatives on the critical functions, generating a first prioritization among risk management initiatives based solely on stakeholders’ priorities. The set of risk management initiatives identified are then evaluated against different possible scenarios with the goal to identify initiatives which are robust across a range of plausible futures (Fig. 2). Different scenarios can describe both climatic (e.g., precipitation or temperature increase) or nonclimatic (e.g., urbanization, population growth) factors, which affect key critical functions and are defined based on the best available knowledge or the diverse view of stakeholders. Based on literature review and expert knowledge, a first assessment of which critical functions would be impacted by each of the considered scenarios is conducted. Then, an assessment of the impact of each selected scenario on critical functions (e.g., low, medium, or high impact) is performed. This influence can be assessed based on available information, using quantitative data or expert judgement. The introduction of scenarios results in a reranking of risk management initiatives which modifies the previous assessment of stakeholders’ preferences.

## 3 RESULTS

In the following sections, the results of the application to the case study of the Metropolitan City of Venice of the scenario‐informed multicriteria methodology presented in Section 2.3 are described.

### 3.1 Critical functions identification and weights assessment

For the Metropolitan City of Venice and its lagoon, the set of critical functions considered was selected in relation with the scope of the analysis, the spatial scale and based on the perspective and priorities of the local stakeholders involved. Selected critical functions belong to five different capital typologies commonly used in climate risk and sustainability assessment (Goodwin, 2003):

- Manufactured capital: material assets or real estate built and human‐made.
- Social capital: factors that constitute human capital at the individual and collective level.
- Cultural capital: tangible artefacts and immaterial aspects of culture.
- Natural capital: natural resources and processes, renewable and nonrenewable, that produce goods and services for human wellbeing.
- Economic capital: various economic sectors, which produce an income and allow the exchange of previous types of capital.

Accordingly, a set of 11 critical functions was selected and summarized in Table II along with the different capitals and with their correspondent description.

**Table II:** Description of the Capital Categories and of the Respective Critical Functions Considered for the Study Area

Capital	Critical Function	Critical Function Description
Manufactured capital	C_1_‐Housing	Urbanized areas of residential type.
	C_2_‐Infrastructures	Transport and communication systems (road network, rail network, airports, and port areas) and energy lines.
Social capital	C_3_‐Population	Population at the census section level, providing information on age, genre, and population density.
Cultural Capital	C_4_‐Cultural sites	Historical centers, museums, and roads of historical‐environmental value representing the historical and cultural heritage of the society and providing a recreational service for resident population and tourists.
Natural Capital	C_5_‐Forests and semi‐natural areas	Wooded areas and seminatural environments particular important for biodiversity conservation and for the provision of various ecosystem services (e.g., regulation of air quality, timber production, etc.).
	C_6_‐Beaches	Beaches and the associated vegetation important for their environmental value linked to the presence of priority habitats, and for the various services they provide, including the recreational bathing one.
	C_7_‐Wetland and Water bodies	Wetlands, an interface environment between the land and aquatic ecosystem, and the hydrographic network. Both are of particular importance for maintaining biodiversity and providing water services.
	C_8_‐Green urban areas	Furnishing greenery (historic gardens, urban parks, neighborhood green spaces, tree‐lined avenues) and functional greenery (for sports, education, health) serving as recreational and leisure areas for the resident population and providing various regulatory services (e.g., air quality regulation in urban areas and rainwater infiltration).
Economic capital	C_9_‐Primary sector	Gross Value‐Added product (GVA) associated to agricultural areas, comprising all the arable areas, the permanent crops, and the pastures.
	C_10_‐Secondary Sector	GVA associated with industrial areas.
	C_11_‐Service sector	GVA of the service sector (e.g., commercial activities, tourism, etc.).

For each critical function, the qualitative relevance value assigned by local stakeholders was converted into a quantitative one (0—*no relevance*, 1—*low relevance*, 2—*medium relevance*, and 3—*high relevance*). The mean value that stakeholders gave to each critical function during the workshop (Section 2.2) was considered as the overall weight to each of them. Resulting scores assigned during the workshop are reported in Table III.

**Table III:** Critical Function Assessment

Critical Functions	Relevance	Weights
c_1_: Housing	High relevance	3
c_2_: Infrastructures	High relevance	3
c_3_: Population	High relevance	3
c_4_: Cultural sites	Medium relevance	2
c_5_: Forests and semi‐natural areas	Medium relevance	2
c_6_: Beaches	Medium relevance	2
c_7_: Wetland and Water bodies	High relevance	3
c_8_: Green urban areas	Medium relevance	2
c_9_: Primary sector	Medium relevance	2
c_10_: Secondary sector	Medium relevance	2
c_11_: Service sector	Medium relevance	2

From Table III, it is possible to observe that housing, infrastructures and population have been weighted with the highest priorities. For these critical functions, stakeholders during the discussion agreed on assign a priority importance independently of their sector of expertise as they are strongly related with human life and well‐being. Wetlands and water bodies were also scored with high relevance as even stakeholders not directly involved in the natural sector recognized their value not only in natural terms (e.g., unique ecosystems and providers of supporting and regulating ecosystem services) but also in their importance as infrastructure in the context of the Venetian Lagoon (e.g., channels for navigation). However, none of the critical functions were weighted with low or no relevance.

### 3.2 Risk Management Initiatives Selection and Assessment

Stakeholders of the Metropolitan City of Venice suggested a set of eleven risk management initiatives, belonging to the different resilience stages and resilience domains (Section 2.3) which are described in Table IV. For each risk management initiative and critical function intersection the qualitative impact value, representing the impact that a specific initiative can have on the critical function, was converted in a quantitative one (0—*low or no impact*, 1—*medium impact*, and 2—*high impact*). Table V summarizes the impact value scores assigned to each critical function of the Metropolitan City of Venice. Importantly, each stakeholder assessed the 11 risk‐management initiatives only against the critical functions belonging to its sector/sectors of expertise (e.g., stakeholders who are expert in natural systems assessed only the critical functions belonging to the natural capital), allowing us to have a more competent judgement. All the scores assigned to an intersection were averaged and subsequently rounded to the nearest integer.

**Table IV:** Risk Management Initiatives Description

Risk Management Initiatives	Risk Management Initiatives Description	Resilience Stage	Resilience Domain
p_1_: Information: common good	Data collection and analysis, information production and sharing in order to develop the knowledge base required to identify climate change impact hotspots, enable climate adaptation, and plan options. Information can include reports, risk and vulnerabilities maps, statistics, climate change projections, knowledge platforms, and networks.	Preparedness, Response	Information
p_2_: Green and blue infrastructures networks	Networks of natural and seminatural landscape elements that build upon ecosystems to meet global challenges, such as risk reduction for storms, landslides, and floods. These solutions include, for instance, the maintenance of vegetated dunes, wetlands, and salt‐marshes restoration, beaches reconstruction, the creation of green corridors in urban and agricultural areas, establishment, and restoration of riparian buffers.	Prevention	Physical
p_3_: Updating and implementation of plans and regulations	Updating and implementing plans and regulations to guide territorial and urban planning, as well as land and natural resource management which play a significant role in risk prevention. Such initiatives include, for instance, limiting the urbanization in flood prone areas, encouraging flood and drought risk‐sensitive land use and management practices, setting the limits for water abstractions, constituting nature and biodiversity protected areas.	Prevention	Cognitive
p_4_: Adaptation and optimization of water infrastructures and supply	Adaptation and optimization of water infrastructures and supply systems to increase their efficiency under water scarcity and drought conditions. Such initiatives include, among others, actions such as water leak control during transport, differentiation of water supply sources, creation of reservoirs, and improvement of irrigation efficiency.	Prevention, Response	Physical
p_5_: Adaptation of hydraulic defense structures	Adaptation or improvement of physical and engineered structures to strengthen and enhance their protection capacities and meet safety requirements under changing conditions (e.g., sea level rise increase, sever flood and storm surge, strong winds extreme temperatures). Such initiatives include the construction or raising of embankments, quaysides, public paved areas, dikes, dams, system of bulkheads located in the Lagoon inlets (e.g., MOdulo Sperimentale Elettromeccanico [MOSE]).	Prevention	Physical
p_6_: Emergency response arrangements	Putting in place physical and structural arrangements to respond to emergencies and limiting the impact of the events on people, materials, and built structures. Onsite initiatives include for instance the installation of temporary footbridges, case, pumps, and mobile bulkheads on private buildings doors.	Prevention, Response	Physical
p_7_: Early warning systems	Enhancing the preparedness of decisionmakers and private individuals to climate‐related natural hazards and their readiness to harness favorable conditions by means of the timely and systematic monitoring, dissemination and communication of relevant information on different types of adverse events. Early warning systems can be developed for flood, high water, heatwaves, and drought and based on different media (e.g., bulletins, mobile Apps, acoustic signals, emails).	Preparedness	Information
p_8_: Environmental education and awareness	Actions promoting the public awareness for the altered conditions under climate change with the final aim to achieve long‐term lasting behavioural changes. These actions can include environmental education programs, awareness campaigns developed through different kind of media and means (e.g., television, internet, newspapers, lectures), and targeting diverse public groups (e.g. children, students, citizens).	Prevention, Preparedness	Social
p_9_: Citizen science	The involvement of citizens, many of whom have no specific scientific training, in scientific research—whether community‐driven research or global investigations. Involved citizens can span over different groups (e.g., students, farmers, fishermen, children, elderly people) and different activities including environmental data collection and observation, passive sensing using sensors installed on mobiles or other devices, participatory sensing where participants actively participate to measurement campaigns (i.e., Acqua Alta Kids Discovery).	Prevention, Preparedness, Response	Social
p10: Civil protection machine planning	Preparation and training of civil protection volunteers, protocols, equipment, and contingency plans to be used in the event of an emergency.	Preparedness, Response	Cognitive
p_11_: Plans and strategies for restoration and recovery of historical areas	Drafting of plans and strategies for restoration and recovery of historical areas, considering conservation needs under future climate scenarios.	Prevention Recovery and Rehabilitation	Cognitive

**Table V:** Assessment of Each Risk Management Initiative with Respect to Each Critical Function

Critical functions Risk Management Initiatives	c_1_: Housing	c_2_: Infrastructures	c_3_: Population	c_4_: Cultural Sites	c_5_: Forests and Seminatural Area	c_6_: Beaches	c_7_: Wetlands and Water Bodies	c_8_: Green Urban Areas	c_9_: Primary Sector	c_10_: Secondary Sector	c_11_: Service Sector
p_1_: Information: common good	1	1	2	2	1	1	2	1	2	2	2
p_2_: Green and blue infrastructures networks	1	1	1	1	2	1	2	1	1	1	1
p_3_: Updating and implementation of plans and regulations	2	2	2	1	1	1	1	1	2	2	2
p_4_: Adaptation and optimization of the water network and supply	1	2	2	1	1	0	2	1	2	1	1
p_5_: Adaptation of hydraulic defense structures	2	2	2	2	1	2	2	1	2	1	0
p_6_: Emergency response arrangements	2	2	2	2	1	1	1	1	1	1	2
p_7_: Early warning systems	2	1	2	2	0	1	1	1	1	2	2
p_8_: Environmental education and awareness	1	1	2	1	2	1	1	1	1	1	1
p_9_: Citizen science	1	1	2	1	1	1	1	1	1	1	1
p_10_: Civil protection machine planning	2	2	2	2	1	1	1	1	1	2	2
p_11_: Plans and strategies for restoration and recovery of historical areas	2	1	2	2	0	0	0	1	0	0	0

Most of the initiatives are considered to have a *medium* or *high* impact on the analyzed critical functions, particularly on population, housing, infrastructures, and cultural sites. On the contrary, the impact scores that they assigned are generally lower for the critical functions belonging to the natural and economic capitals, for which, in fact, different initiatives are also scored with *no or low* impact. These results can be explained by the fact that stakeholders belonging to natural and economic capitals confirmed, also in the assessment of the risk management initiatives, the priority importance that they give to the human capital. In addition, although many stakeholders belonged to the natural sector of expertise, not a lot of risk management initiatives specifically targeted for such capital were suggested and thus were available for them to evaluate with high scores.

### 3.3 Description of Scenarios and Impact on Critical Functions

In the workshop, a set of climate change scenarios was proposed by the experts and discussed together with stakeholders in order to identify the most representative ones for the case study area. The final set of scenarios, identified as the most prominent by local stakeholders, are:

- *S*_1_: increase frequency of storm surge events.
- *S*_2_: increase frequency of pluvial flood
- *S*_3_: increase frequency of heat waves.
- *S*_4_: increased frequency of drought conditions.

The selected narrative climate change scenarios were then characterized by experts by a set of Climate Extreme Indices (CEIs), commonly used as “proxies” for hazards characterization (Mysiak et al., 2018), and derived from the outputs of Regional Climate Models (RCMs) available for the case study for the future medium term period (i.e. 2021–2050) considering the Representing Concentration Pathway (RCP) 8.5. For more details about CEIs used see the Appendix II of Supporting Information (Table A1).

The degree of impact (low, medium, or high) that each climate change scenario would have on each of the critical function has been assessed based on the results of a climate change risk study performed by Sambo (2020) (Table VI). If a critical function was considered not to be impacted by a specific climate change scenario the degree of impact has not been assessed and this result as a blank spot in Table VI. The qualitative impact values of the climate change scenarios on the critical functions were then converted into quantitative ones (1—no impact, 3—low impact, 5—medium impact, and 7—high impact). These values are used as *α* multiplier that increases the weight of a critical function under a specific climate change scenario (Equation A2, Supporting Information, Appendix I). These scores were finally used to perform Equation A3 (Supporting Information, Appendix I) obtaining in this way a new climate change scenario‐based ranking of risk management initiatives.

**Table VI:** Impact Classification for the Scenarios Considered on Each Critical Function

	s_1_: Storm Surge	s_2_: Pluvial Flood	s_3_: Heat Waves	s_4_: Drought
c_1_: Housing	High Impact	Medium Impact		
c_2_: Infrastructures	High Impact	Medium Impact		
c_3_: Population	Medium Impact	Low Impact	Low Impact	Medium Impact
c_4_: Cultural sites	High Impact	Medium Impact		
c_5_: Forests and semi‐natural areas	Medium Impact			
c_6_: Beaches	High Impact			
c_7_: Wetlands and water bodies	High Impact			
c_8_: Green urban areas	High Impact			
c_9_: Primary sector	High Impact			Low Impact
c_10_: Secondary sector	Low Impact	Medium Impact		Medium Impact
c_11_: Service sector	Medium Impact	Low Impact	Low Impact	

The results show how increased frequency of storm surge events is affecting all the critical functions, and mainly with a *high* impact. The increased frequency of pluvial flood is going to affect (with *medium* or *low* impact) all the critical functions characterized by artificial and, thus, nonpermeable surfaces, which are therefore more pressured by heavy precipitation events. The increased frequency of heat waves is going to affect only the human components of the coastal system, for which is expected a low impact. Finally, the increased frequency of drought conditions is going to affect population and secondary sector, which are both expected to be medially impacted. Drought conditions are going to slightly affect (i.e., *medium* impact) also the primary sector.

### 3.4 Risk Management Initiatives Prioritization

The use of the scenario‐based multicriteria methodology described in Section 2.3 allowed a ranking of the 11 risk management initiatives previously identified (Table IV) by stakeholders as the most prominent ones for the case study area. The ranking is to be intended as the order of the initiatives in term of their effectiveness in improving the overall resilience of the analyzed system.

**Fig 4:**
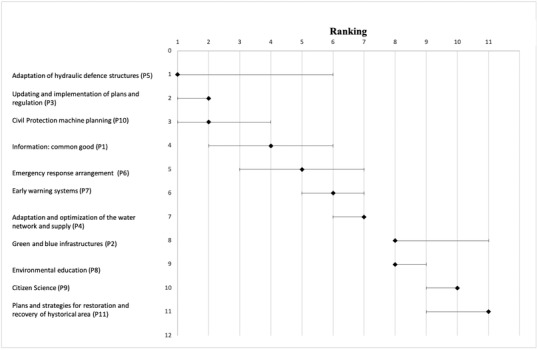
Risk management initiatives baseline rankings (diamonds) and the ranges of rankings associated to the four climate change scenarios (horizontal bars)

In Fig. 4, diamonds display the baseline ranking of the risk management initiatives. As explained in Section 2.3, the baseline ranking is calculated taking only into consideration values scores that stakeholders gave to the critical functions and the scores that they assigned to the impact of the initiatives on critical functions. Accordingly, the baseline ranking does not consider the effect of climate change scenarios. Among the top five positions we can find initiatives belonging both to the physical (i.e., *Adaptation of hydraulic defence structures [P5]* and *Emergency response arrangements [P6])*, cognitive (i.e., *Civil Protection machine planning [P10] and Updating ad implementation of plans and regulations [P3]*), and information (i.e., *Information: common good [P1]*) domains. Considering the resilience stages, the initiatives placed in the top five positions are dealing mainly with prevention (i.e., *P3, P5*, and *P6*) and with preparedness and response stages (i.e., *P10, P1*).

The last positions, instead, are occupied by nature‐based solutions (i.e., *Green and blue infrastructures [P2]*), social initiatives (i.e., *Environmental education [P8], Citizen science [P9])*, and a very specific cognitive initiative dealing with historical and cultural heritage sites (*i.e., Plans and strategies for restoration and recovery of historical area [P11])*. Regarding the resilience stages, the initiatives in the last positions are distributed among all the stages, also because various of these initiatives fall themselves into different resilience stages.

The horizontal bars associated to each diamond in Fig. 4 show the ranges of rankings that each risk management initiative can take due to the integration of the four additional climate change scenarios into the assessment. Independently from the position in the baseline, a small range bar means that an initiative is stable across the different climate change scenarios, on the contrary a large range bar means that an initiative widely changes its position depending on the considered scenario. Most stable initiatives are represented by *updating and implementation of plans and regulations* (P3), *adaptation and optimization of the water network and supply* (P4), *environmental education and awareness* (P8), *citizen Science* (P9). *adaptation of hydraulic defense structures* (P5), *information common good* (P1), and *emergency Response arrangements* (P6) instead largely vary their position when climate change scenarios is introduced.

Rankings of initiatives are quite different when one climate change scenario at time is considered. Table VII shows risk management initiatives rankings for the baseline, for a single climate change scenario and across all the scenarios. Looking at the physical initiatives, we can see that *adaptation of hydraulic defense structures* (P5), which in the baseline is on top, stays on top positions for storm surge's scenario but it drops positions when considering pluvial flood, heat waves, and drought events (it drops from position 1 to position 4, 6, and 3, respectively). In the same way, *emergency response arrangements* (P6) slightly advances in the ranking (from position 5 to positions 4, 3, and 4 respectively) when considering the storm surge, pluvial flood, and heat waves scenarios individually; however, it drops in position when considering just drought. On the contrary, *adaptation and optimization of the water network and supply* (P4), slightly advances in the ranking considering the drought scenario. *Green and blue infrastructures (P2)* is already in a low position, and it drops further for pluvial flood, heat waves, and drought. Looking at cognitive and informative initiatives we can see how *information: common good (P1*), *updating and implementation of plans and regulations (P3)*, and *Civil protection machine planning (P10)* remain in a high‐ranking position across almost all the considered scenarios. *Early warning systems (P7)* is already quite stable and always positioned in the middle of the rankings. Even the initiatives belonging to the social domain, such as *Environmental education (P8)* and *Citizens science (P9)*, remain quite stable for all the four scenarios, ranging from position 8 to 10, never appearing among the priority ones. *P8* drops only for storm surge from position 8 to 9, while *P9* rises from 10 to 9 for all the scenarios except for storm surges, where it remains at the same position.

**Table VII:** Risk Management Initiatives Ranking for the Baseline, for Single Climate Change Scenarios and Across All the Scenarios

	Ranking					
Ordered Risk Management Initiatives	Baseline	Storm Surge	Pluvial Flood	Heat Waves	Drought	Multiple Scenarios
Adaptation of hydraulic defense structures (P5)	1	1	4	6	3	3
Updating and implementation of plans and regulations (P3)	2	2	2	1	1	1
Civil Protection machine planning (P10)	2	2	1	1	4	2
Information: common good (P1)	4	5	6	3	2	4
Emergency response arrangements (P6)	5	4	3	4	7	5
Early warning systems (P7)	6	7	5	5	5	6
Adaptation and optimization of the water network and supply (P4)	7	6	7	7	6	7
Green and blue infrastructures networks (P2)	8	8	11	10	10	10
Environmental education and awareness (P8)	8	9	8	8	8	8
Citizen Science (P9)	10	10	9	9	9	9
Plans and strategies for restoration and recovery of historical areas (P11)	11	11	9	11	11	11

When considering multiple scenarios, the overall ranking differs from the ones obtained considering single scenarios individually. Comparing the overall ranking with the baseline one, few shifts in positions can be observed as *updating and implementation of plans and regulation (P3)* moves in first position replacing a rather strong physical initiative, *adaptation of hydraulic defense structures (P5), green and blue infrastructures networks (P2)*, and *citizen science (P9)* are reversed in the ranking.

### 3.5 Scenario Disruptiveness

Together with the prioritization of the risk management initiatives, an assessment of the scenarios’ influence on stakeholders’ priorities was performed. The calculation of disruption scores is described by Thorisson et al. (2017) and Schroeder and Lambert (2011). Understanding the disruptiveness of the climate change scenarios will help stakeholders in the determination of project initiatives to discuss given the scenarios of greater concern (Bostick et al., 2017). Some climate change scenarios may have very little impact on the risk management initiatives ranking in the baseline scenario, but others may completely change the prioritization. This effect was captured by the scenario's disruptiveness metrics (Equation A4, Supporting Information, Appendix I). Table VIII displays the sum of squared error scores for the four considered scenarios. A higher sum of squared error scores would indicate that a particular scenario has increased influence on changing risk management initiatives’ ranking. Therefore, the scenarios are classified according to their degree of influence on the ranking of project initiatives based on the sum of square error scores.

**Table VIII:** Sum of Squares Error Values for Quantifying the Disruptiveness of the Four Scenarios

Scenario	SSE
Heat waves	35
Pluvial flood	33
Drought	24
Storm surges	5

The most influential scenario according to this disruptiveness metric is the heat waves. When comparing the priority orders of the initiatives under this scenario and under the baseline scenario, changes in the ranking occur for eight out of 11 initiatives. While most of them are characterized by small shifts in the ranking (less or equal to two positions), significant changes (more o equal to three position) occur for *adaptation of hydraulic defense structures (P5)*, which moves from position 1 to position 6. Nine out of 11 initiatives change their position in the drought scenario when comparing with the baseline one, but none of them are characterized by significant alterations. Instead, low metric scores characterize storm surge, due to small shifts in the ranking for only four initiatives out of 11.

**Fig 5:**
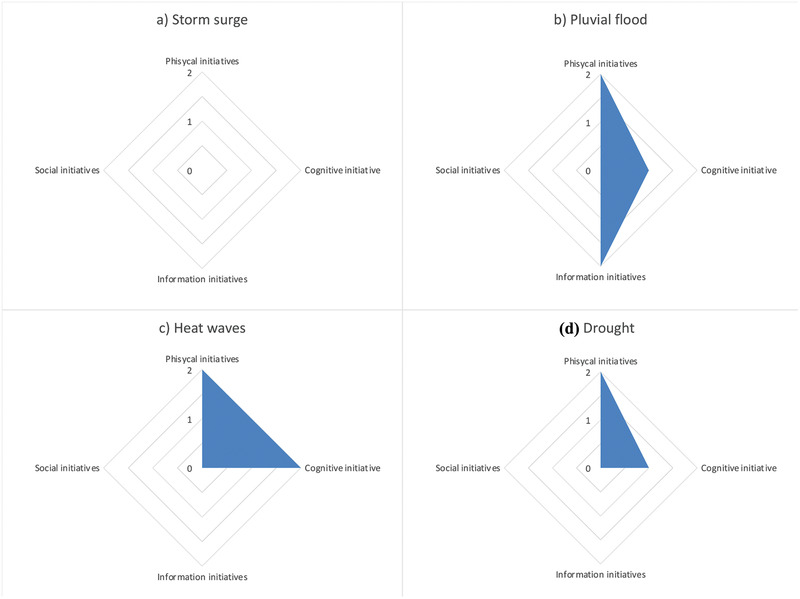
Disruptiveness of scenarios: (a) storm surge, (b) pluvial flood, (c) heat waves and (d) drought (with diagrams adapted from Lambert et al. (2013) and You, Connelly et al. (2014), and You, Lambert et al. (2014).

We can observe the same trend when analyzing the disruptiveness of the scenarios separately on the ranking of initiatives belonging to different domains (Fig. 5). According to this metric storm surge scenario is not disruptive at all, as it does not change the position of any of the initiatives in the four domains. This can be explained by the fact that storm surge is already perceived as the most severe threat in Venice, taking into account also the exceptional high water events occurred in these last years, and for this reason, the measures have been proposed with this perception and specifically with the aim of cope with this hazard. The drought scenario influences the change on the ranking position of the initiatives belonging to the physical and cognitive domains, while the pluvial flood scenario the ones belonging to the physical, cognitive, and information domain. Heat waves influence the change on the ranking position of the initiatives belonging to physical and cognitive domains. Heat waves and pluvial flood are the most disruptive scenarios, which results, in fact, with the largest areas in the graph (Fig. 5).

## 4 DISCUSSION

The above results suggest how stakeholders have varying perspectives and priorities for adaptation and resilience. Such preferences indirectly reflect on the choice and ranking of the risk management initiatives. The positions of risk management initiatives in the baseline ranking depend on their impact on the critical functions and on the relevance stakeholders assigned to that particular critical function. Initiatives appearing in the top five positions, in fact, are those providing most beneficial effects on the manufactured (i.e., housing, infrastructures) and social (i.e., population) capital, priority critical functions for the case study according to stakeholders. Many of the risk management initiatives that appear in the top positions (Table VII) have a positive impact also on the cultural and economic sectors, although these sectors were initially underrepresented among the involved stakeholders (Table I).

Moreover, the workshop format allowed for an open exchange between the involved local actors enabling a support between each other in emphasizing a certain shared priority or a mitigation of their positions. The opportunities to share, among stakeholders and between stakeholders and the experts, knowledge, and expertise and to enable learning provided by the workshop format, are recognized to be necessary to build effective disaster risk management (O'Brien, O'Keefe, Gadema, & Swords, 2010).

Climate scenarios substantially influence the ranking of risk management initiatives and disrupt stakeholders’ priorities. However, such changes can be more or less pronounced, depending on the stability of selected initiatives to different scenarios. The results suggest how initiatives belonging to the physical domain, despite being in top positions, are generally less stable. These results can be explained by the fact that physical initiatives are usually designed and implemented targeting very specific typologies of extreme climate events (i.e., storm surge, flood, etc.) (Royal Society, 2014). For example, the design of *hydraulic defense structures* and the implementation of *emergency response arrangements* including a set infrastructural projects like the MOSE – (MOdulo Sperimentale Elettromeccanico) or temporary solutions (e.g. footbridges, pumps and mobile bulkheads on private buildings doors) are specifically designed for the protection of the Metropolitan City of Venice from storm surge and high waters events, while lacking any ability to increase the system resilience in relation with other kinds of hazard (i.e. drought, heatwaves). The triggering flood threshold for raising the MOSE barrier is a negotiated level involving maritime commerce and social factors, such that frequent low‐level floods will still occur in the Metropolitan City of Venice. Also, physical initiatives adopting a nature‐based approach (e.g., the implementation of green and blue infrastructure networks) are quite unstable. As recalled by Calliari, Staccione, and Mysiak (2019) nature‐based solutions are “living” solutions whose effectiveness is determined both by the magnitude of the threats, as well as their ability to adapt to environmental and anthropogenic pressures to which they are exposed. Climate change, in particular, can alter ecosystems and their services, and may undermine the performance of green and blue solutions relying on them (Seddon et al., 2020).

On the contrary, cognitive, informative, and social initiatives, seem to be more stable under changing conditions, as they maintain their position when climate change scenarios are introduced. Such initiatives, as opposed to physical ones, are not aimed at coping with specific hazard typologies but rather they are thought to increase the overall resilience of the system across different possible adverse events exploiting the power of institutions, the sharing of knowledge and the public involvement. Initiatives related with implementation of plans and regulations (e.g., P3) as well as based on the use of early warning systems (e.g., P7), acting on the way that people perceive and react to extreme events, no matter of which type they are, are prone to promote behavioural changes that, on long term horizon, have greater potential to enhance resilience overall (IPCC, 2012).

Also, initiatives directly involving citizen in scientific research, data collection, and observations (i.e., Acqua Alta Kids Discovery) gain position relative to the baseline suggesting that such kind of initiative becomes fundamental when climate change scenarios come in place. Recent reports from both IPCC (2014)and Royal Society (2014) confirm that social approaches are vital in building resilience as raising the awareness and knowledge of specific community groups toward climatic phenomena make them also more supportive toward other kind of adaptation options (Tompkins & Adger, 2004).

Different climate scenarios, when analyzed one by one, lead to a specific prioritization of the set of risk management initiatives. At the same time, when the scenarios are analyzed together, the prioritization of the same initiatives can be totally different.

However, given the large uncertainty in predicting which hazard scenarios may occur in a particular area, the best option is to build overall resilience of coastal systems in the face of a range of adverse events. For this reason, it is fundamental to select initiatives which are optimal from a multihazard perspective. Results suggest that, in the Metropolitan City of Venice, options suitable to cope with multiple hazards are cognitive (i.e., *updating and implementation of plans and regulation [P3]*) and social ones (i.e., *citizen science* [P9]). Both of them, in fact, gain positions in the ranking, and the former one especially replaces a rather strong physical initiative (i.e., *adaptation of hydraulic defense structures* [P5]) at the top position. As demonstrated by the Royal Society (2014), physical initiatives have the lowest potential to adapt to multiple adverse events and thus nonphysical initiatives are preferable in a context of high uncertainty as the one induced by climate change.

Heat waves was the scenario with the highest potential to disrupt stakeholders’ priorities while storm surge was the least influential one. Although, climate change scenarios for the case study have been introduced to stakeholders only at a late stage, it must be considered that they may have already experienced climate change effects and it tends to influence their decisions (Bronfman, Cisternas, Repetto, Castañeda, & Guic, 2020) and, indirectly, the selection and ranking of risk management initiatives. In the Metropolitan City of Venice, given the intensity of the high‐water events occurred in the last years, storm surge is in fact perceived as the most severe threat. Accordingly, most of the initiatives which have been proposed are targeting this hazard. This vision was explored in the workshop with local stakeholders, stressing the importance of providing decisionmakers with relevant information to overcome their biases and to select strategies for resilience, maximizing efficiency and economic efforts given a set of scenarios.

## 5 CONCLUSIONS

In this article, a scenario‐informed multicriteria methodology to support the analysis and prioritization of risk management initiatives aimed at enhancing resilience towards multiple climate related stressors was developed. The methodology was applied and tested to the case study of the Metropolitan City of Venice in Northern Italy considering several representative scenarios of climate‐related extremes (e.g., storm surges, pluvial floods, heat waves, drought) that could impact different coastal systems and functions (i.e., natural, cultural, social, and economic).

A feature of this analysis is the integration of top‐down information, quantitative data derived from regional climate change model projections and impact analysis, together with a bottom‐up qualitative information elicited from local actors and stakeholders in a workshop, into a final tailored framework for resilience assessment. Various stakeholders were involved across sectors of expertise and fields of interest; these include national, regional, and local authorities, independent authorities, research institutions, parks, and NGOs. Stakeholders identified the priorities and necessities for the resilience‐enhancing initiatives. On the other hand, they have been presented to different climate change scenarios, having the opportunity to improve their wealth of knowledge about the most prominent hazards for the case study area.

Independently from their sector of expertise, stakeholders agree on assigning a priority importance to critical functions belonging to manufactured (i.e., housing, infrastructures) and social (i.e., population) capital, as they are strongly related with human life and well‐being. As a consequence, risk management initiatives that appear in the top five positions in the baseline ranking are those that have a major impact on such critical functions. These are *adaptation of hydraulic defense structures (P5), emergency response arrangements (P6), civil protection machine planning (P10)*, *updating ad implementation of plans and regulations (P3)*, and *information: common good (P1)*, which belong to the physical, cognitive, and information domains. The use of scenario‐based preferences allows understanding of how each of the four considered climate‐change scenarios (i.e., storm surge, pluvial flood, heat waves, and drought) is prone (or not) to disrupt stakeholders’ priority for risk management. For all scenarios, initiatives belonging to the physical domain are generally less stable, while cognitive, informative, and social initiatives seem to be more stable under changing conditions. However, when comparing the priority orders of the risk management initiatives under the heat wave scenario and under the baseline scenario, changes in the ranking occur for a higher number of initiatives (eight out of 11) with respect to changes in the ranking under other scenarios. According to the disruptiveness metric, heat waves is, in fact, the most influential scenario.

From the results also emerge that different scenarios, when analyzed one by one, lead to a specific prioritization of the set of risk management initiatives; meanwhile, when the scenarios are analyzed together, the prioritization of the same initiatives can be totally different. The necessity to adopt a multihazard approach to disaster risk management and climate change adaptation is confirmed by these outcomes. In fact, the initiatives preferred to increase the overall resilience considering more than one scenario can be more efficient in case of high uncertainty than sectorial initiatives that are targeted for a specific hazard. Implementing initiatives strongly oriented to cope with single hazard could lead to an increase of a risk toward other kind of hazards (i.e., maladaptation) thus undermining efforts and resources invested for risk reduction. Accordingly, a portfolio of risk‐management initiatives should be used to enhance the resilience of the system including physical initiatives to cope with large scale and intense events together with cognitive and social ones which can be flexible enough to be effective against a range of hazards.

In future analyses, we recommend iteration by addressing how the stakeholders’ preferences for receptors and initiatives might change. It must be considered that stakeholders may have prior knowledge and perceptions of some specific hazards that could occur in the case study area, and this could have influenced the score allocation; in fact, they could have some *a priori* ideas on climate change future scenarios. It can be seen from the workshops results: most of involved stakeholders perceived storm surge as the most severe threat for the case study area and in fact most of the proposed risk management initiative proposed are specifically designed to cope with this kind of hazard.

The approach of this effort could be improved considering additional terrestrial, coastal, and marine climate‐related extreme and scenarios (e.g., water quality alteration, river flooding, sea level rise, increase sea surface temperature). Moreover, socioeconomic scenarios describing the future evolution of socioeconomic dynamics (e.g., urbanization, population growth, migrations, tourism) could be integrated to take into account the effect of their interaction with climatic drivers in exacerbating the risk and vulnerability towards disasters. This could be done by integrating outputs derived from land use change model, economic models, and demographic projections. At the same time, the methodology can be enriched included additional critical functions (i.e., electric network, navigation channel, migrants, fishing valleys, hydric resources, resident population) to better describe site‐specific peculiarities and processes as suggested by stakeholders during the workshop.

A key outcome of the proposed methodology is an identification of scenarios that are most and least disruptive to the prioritization of risk management initiatives; such understanding contributes to overall resilience of the coastal systems. It is an essential piece of knowledge in the analysis of the resilience of the Metropolitan City of Venice to climate‐related and other factors including the current pandemic (i.e., COVID‐19), over‐tourism and depopulation. These outputs can then be used to support the implementation of climate change adaptation and disaster risk reduction policies and strategies. The participative process initiated within the BRIDGE project represent a step towards the establishment of a community of practice for disaster risk reduction and climate change adaptation in the Metropolitan City of Venice: through the workshops, diverse actors involved in the resilience building cycle had the opportunity to sit at the same table and to discuss to reach agreement on a shared set of measures to be implemented. Most of actors involved were interested in staying engaged in future steps of the process, giving room to the hope that the cooperation exercise performed during the workshop could be reflected in the reality in the implementation of a shared local plan for climate change adaptation.

Some of the initiatives proposed for prioritization were already part of existing plans (i.e., geo‐morphological plan of the Venice lagoon) or derived from implemented adaptation projects and pilots (i.e., LIFE Seresto, LIFE Vimine). Testing their efficiency toward a set of multiple scenarios, provided practitioners with new insights and recommendations to be considered for future efforts, like for instance, adopting a multihazard perspective to DRR and the need for a stronger inter‐sectoral coordination in climate change adaptation.

## Supporting information



Supplementary Material
