# Supplementary material for: Prioritization of Resilience Initiatives for Climate‐Related Disasters in the Metropolitan City of Venice
Source: Risk Anal. 2021 Sep 17;42(5):931–52. doi: 10.1111/risa.13823 (PMC9544050; doi:10.1111/risa.13823)
Supplement: Supplementary file 1 — Supplementary Material [file RISA-42-931-s001.docx]

**ANNEX I**

The first step of the methodology (Fig. 2) requires the identification of key critical functions (i.e. sub-systems and processes that are likely to be affected by extreme events) for the system under analysis, that will form the basis for comparing project initiatives. Critical functions can be represented by physical, environmental, cultural or socio-economic sub-systems and processes whose protection is crucial to maintain and enhance the overall resilience of the system.

A total of N critical functions is selected, and this produces a set of vector-valued critical functions, C, for the system taken into account, where:

C= {c1, c2, . . ., cN}.

Later, the relative importance of the *c*^th^ critical function C_N_ within the system is provided and assessed through the relevance function *w_c._* The relevance value score (w_c_), represents mathematically the value judgements of stakeholders and it is used for the prioritization of critical functions within the systems. High values of w_c_ imply a higher priority of that particular critical function. The selected form of the value function is incidental to the methodology and therefore special attention should be payed to this step of the assessment.

In the second step (Fig. 2), a set of risk management measures beneficial in enhancing the overall resilience of the system to climate-related extreme events have to be selected and proposed for prioritization. Risk management measures can involve the allocation of resources, policies, structural and non-structural interventions encompassing different risk management stages as described (Fig. 3, Section 1.3). Therefore, a set of N initiatives, P, is considered and proposed for prioritization:

P= {p1, p2, . . ., pN}.

Even in this step, the risk management measures are chosen considering those most beneficial for the particular case study analysed taking into account stakeholders’ perspectives and needs.

Later, for each risk management measure identified (*p_i_*), the impact (no impact, low, medium, high) on each of the c_N_ critical function is assessed by assigning an impact value score (v_c_(p_i_)). The score *v_c_(p_i_),* that is the assessed value of project initiative *p_i_* on the critical function c_N_, indicates how much the project initiative could impact the critical function. If *v_c_(p_i_)* is *zero*, it means that the project initiative taken into account has low or no impact on that particular critical function, while a higher value means that the measure has a significant impact on the critical function.

The relevance value score (Equation A1) generates a first prioritization among risk management measures.

$V\left( p_{i} \right)=\sum_{c=1}^{C} w_{c}v_{c}(p_{i})$ Equation A1

where:

V(p_i_) represents the value score of the risk management measure pi;

w_c_ represents the weight assessing the relative importance of the critical function C_N_**;**

v_c_(p_i_) represents the score assessing the impact of the risk management measure on the critical function C_N._

Based on Equation A1, for each risk management measure *p_i_* a value score *V(p_i_)* is assigned thus forming the basis for prioritization of measures such that *p_i_* is preferred to *p_j_* if and only if *V(p_i_)* $>$ *V(p_j_)*; this implies indifferences among *p_i_* and *p_j_*, (*p_i_* $\sim$ *p_j_*) if and only if *V(p_i_)= V(p_j_)*.

The set of P risk management measures identified are then evaluated against different possible scenarios related to extreme events with the goal to identify measures which are robust across a range of plausible futures. A set of scenarios, S, is then defined:

S= {s_1_, s_2_, . . ., s_N_}.

Later, an assessment of the impact of each selected scenario on critical functions is performed. This influence (e.g. no, small, or large increase or no, small, or large impact) can be assessed, based on available information, using quantitative data or expert judgement. Impacting critical functions, the introduction of scenarios is likely to results in a re-ranking of risk management initiatives modifying stakeholders’ preferences previously assessed (Equation A1). For each scenario, *s_j_∈S*, which modifies preferences, the initial relevance value scores of the critical functions are increased based on the impact of the scenario on the critical functions in comparison to the baseline situation (i.e. non scenario considered). To implement this increase in the relevance value scores for the critical functions, a multiplier constant α>1 was used, defining in this way a new nonnormalized coefficient for the *c*^th^ critical function:

*w’_c_=*α x *w_c_*. Equation A2

The interpretation of α criterion, compared to the other ranking previously determined (Equation A1), is that it determines a new weight of the critical function under the new scenario compared to the same ranking under the baseline scenario.

It is now possible to calculate for each risk management measures a new value score *V(p_i_)* that consider also the influence of a specific scenario on the critical functions and that represent a new prioritization of the risk management measures.

$V\left( p_{i} \right)=\sum_{c=1}^{C} w_{c}\alpha v_{c}(p_{i})= \sum_{c=1}^{C} {w'}_{c}v_{c}(p_{i})$ Equation A3

In order to define how the scenarios affect decision- making, risk analysis requires metrics to rank those scenarios according to their degree of influence. This determination can be made by applying Equation A4, where the sum of squares has been used as a metric of disruptiveness, *m(s_j_).* Some scenarios may have a little impact on the ranking, while others may completely change it.

$m\left( s_{j} \right)=\sum_{p=1}^{P} \left( r_{j0}-r_{ij} \right)^{2}$ Equation A4

Where:

r_jo_ represents the prioritised ranking of measures in the baseline scenario

r_ij_ is the ranking value in the j^th^ scenario.

**ANNEX II**

In Table AI are reported the CEIs used to characterise the hazards presented in this study; in particular, the increase of extreme sea level (i.e. medium sea level combined with tides and waves) was used to characterise the increase frequency of storm surge events, the increase of occurrence of days with heavy precipitations was used to characterise the increase frequency of pluvial flood, the increase of occurrence of days with extremely high temperatures was used to characterise the increase frequency of heat waves, while the increase of occurrence of consecutive dry days and the decrease of cumulative summer precipitation were used to characterise the increase frequency of drought conditions.

Table A1: Selected scenarios and correspondent Climate Extreme Indices (CEI) used for its characterization in the Metropolitan city of Venice and its lagoon

| Scenarios | CEI | Long name | Description | Unit | Source |
| --- | --- | --- | --- | --- | --- |
| Flood | ESL | Extreme Sea Level | Extreme Sea Level derived from the combined effect of relative sea level rise, tides, and water level fluctuations due to climate extremes | m | LISCOAST (JRC) |
| Pluvial flood | R95p | Very wet days | Number of days with daily rainfall above the 95th percentile | days | COSMO-CLM (CMCC) |
| Heat waves | SU95p | Summer days | Number of days with maximum daily temperature above 95th percentile | days | COSMO-CLM (CMCC) |
| Drought | CDD | Consecutive dry days | Maximum number of consecutive days with daily precipitation <1mm | days | COSMO-CLM (CMCC) |
|  | SP | Cumulative summer precipitation | Cumulative precipitation in the summer months (June, July, August) | mm | COSMO-CLM (CMCC) |
